# Supplementary material for: Modeling glioblastoma heterogeneity as a dynamic network of cell states
Source: Mol Syst Biol. 2021 Sep 16;17(9):e10105. doi: 10.15252/msb.202010105 (PMC8444284; doi:10.15252/msb.202010105)
Supplement: Supplementary file 5 — Source Data for Figure 3 [file MSB-17-e10105-s001.zip › Figure3A_sourcedata/GSEA_3065/hallmarks_state3.GseaPreranked.1623416274744/HALLMARK_FATTY_ACID_METABOLISM.html]

Details for gene set HALLMARK\_FATTY\_ACID\_METABOLISM[GSEA]

|  || Dataset | state3 |
| Phenotype | NoPhenotypeAvailable |
| Upregulated in class | na\_neg |
| GeneSet | HALLMARK\_FATTY\_ACID\_METABOLISM |
| Enrichment Score (ES) | -0.335964 |
| Normalized Enrichment Score (NES) | -1.2070562 |
| Nominal p-value | 0.15749235 |
| FDR q-value | 0.27006745 |
| FWER p-Value | 0.994 |
Table: GSEA Results Summary

  

Fig 1: Enrichment plot: HALLMARK\_FATTY\_ACID\_METABOLISM      
 Profile of the Running ES Score & Positions of GeneSet Members on the Rank Ordered List

  

| PROBE | GENE SYMBOL | GENE\_TITLE | RANK IN GENE LIST | RANK METRIC SCORE | RUNNING ES | CORE ENRICHMENT || 1 | HSP90AA1 |  |  | 60 | 0.402 | 0.0269 | No |
| 2 | OSTC |  |  | 118 | 0.325 | 0.0476 | No |
| 3 | ERP29 |  |  | 169 | 0.300 | 0.0670 | No |
| 4 | RDH11 |  |  | 212 | 0.277 | 0.0854 | No |
| 5 | DHCR24 |  |  | 244 | 0.265 | 0.1042 | No |
| 6 | HMGCS1 |  |  | 267 | 0.254 | 0.1230 | No |
| 7 | PTPRG |  |  | 268 | 0.254 | 0.1443 | No |
| 8 | ELOVL5 |  |  | 450 | 0.209 | 0.1410 | No |
| 9 | FASN |  |  | 503 | 0.198 | 0.1517 | No |
| 10 | SERINC1 |  |  | 530 | 0.194 | 0.1650 | No |
| 11 | HSPH1 |  |  | 937 | 0.144 | 0.1302 | No |
| 12 | HSD17B11 |  |  | 1083 | 0.132 | 0.1245 | No |
| 13 | HSD17B4 |  |  | 1193 | 0.122 | 0.1222 | No |
| 14 | NSDHL |  |  | 1344 | 0.112 | 0.1143 | No |
| 15 | SDHA |  |  | 1487 | 0.104 | 0.1066 | No |
| 16 | ACOX1 |  |  | 1544 | 0.101 | 0.1086 | No |
| 17 | FH |  |  | 1813 | 0.086 | 0.0848 | No |
| 18 | HIBCH |  |  | 1943 | 0.079 | 0.0765 | No |
| 19 | CPOX |  |  | 2044 | 0.074 | 0.0712 | No |
| 20 | GCDH |  |  | 2092 | 0.072 | 0.0718 | No |
| 21 | IDI1 |  |  | 2285 | 0.065 | 0.0551 | No |
| 22 | IDH1 |  |  | 2376 | 0.062 | 0.0499 | No |
| 23 | GPD2 |  |  | 2606 | 0.053 | 0.0279 | No |
| 24 | HSDL2 |  |  | 2688 | 0.050 | 0.0228 | No |
| 25 | ACAT2 |  |  | 2779 | 0.048 | 0.0164 | No |
| 26 | DLD |  |  | 2811 | 0.047 | 0.0167 | No |
| 27 | UGDH |  |  | 2879 | 0.045 | 0.0127 | No |
| 28 | NBN |  |  | 3008 | 0.041 | 0.0014 | No |
| 29 | ADIPOR2 |  |  | 3051 | 0.040 | -0.0001 | No |
| 30 | PDHA1 |  |  | 3126 | 0.038 | -0.0055 | No |
| 31 | HADHB |  |  | 3282 | 0.034 | -0.0206 | No |
| 32 | MLYCD |  |  | 3359 | 0.032 | -0.0267 | No |
| 33 | EPHX1 |  |  | 3598 | 0.026 | -0.0521 | No |
| 34 | ACADVL |  |  | 3600 | 0.026 | -0.0501 | No |
| 35 | SUCLA2 |  |  | 3648 | 0.024 | -0.0534 | No |
| 36 | HSD17B7 |  |  | 3704 | 0.023 | -0.0579 | No |
| 37 | CPT2 |  |  | 4059 | 0.014 | -0.0977 | No |
| 38 | D2HGDH |  |  | 4111 | 0.012 | -0.1026 | No |
| 39 | NCAPH2 |  |  | 4170 | 0.011 | -0.1084 | No |
| 40 | METAP1 |  |  | 4179 | 0.010 | -0.1085 | No |
| 41 | RAP1GDS1 |  |  | 4374 | 0.005 | -0.1305 | No |
| 42 | APEX1 |  |  | 4599 | -0.001 | -0.1563 | No |
| 43 | MDH1 |  |  | 4655 | -0.003 | -0.1624 | No |
| 44 | ENO2 |  |  | 4676 | -0.003 | -0.1645 | No |
| 45 | GSTZ1 |  |  | 4866 | -0.008 | -0.1857 | No |
| 46 | HMGCL |  |  | 5049 | -0.012 | -0.2057 | No |
| 47 | ADSL |  |  | 5187 | -0.015 | -0.2203 | No |
| 48 | BCKDHB |  |  | 5245 | -0.017 | -0.2254 | No |
| 49 | CA2 |  |  | 5274 | -0.018 | -0.2272 | No |
| 50 | BLVRA |  |  | 5364 | -0.020 | -0.2358 | No |
| 51 | HADH |  |  | 5434 | -0.022 | -0.2419 | No |
| 52 | SDHD |  |  | 5493 | -0.024 | -0.2466 | No |
| 53 | NTHL1 |  |  | 5515 | -0.025 | -0.2470 | No |
| 54 | HCCS |  |  | 5576 | -0.026 | -0.2517 | No |
| 55 | BPHL |  |  | 5635 | -0.028 | -0.2560 | No |
| 56 | ACSL4 |  |  | 5729 | -0.031 | -0.2642 | No |
| 57 | ACOT8 |  |  | 5851 | -0.034 | -0.2753 | No |
| 58 | ALAD |  |  | 5878 | -0.035 | -0.2753 | No |
| 59 | PPARA |  |  | 5879 | -0.035 | -0.2724 | No |
| 60 | ACO2 |  |  | 5970 | -0.038 | -0.2796 | No |
| 61 | GABARAPL1 |  |  | 5976 | -0.039 | -0.2769 | No |
| 62 | IDH3G |  |  | 6007 | -0.040 | -0.2770 | No |
| 63 | ACAA1 |  |  | 6069 | -0.042 | -0.2805 | No |
| 64 | KMT5A |  |  | 6083 | -0.042 | -0.2785 | No |
| 65 | ACSL1 |  |  | 6134 | -0.044 | -0.2806 | No |
| 66 | ACADM |  |  | 6280 | -0.049 | -0.2932 | No |
| 67 | ALDH9A1 |  |  | 6304 | -0.050 | -0.2917 | No |
| 68 | CCDC58 |  |  | 6395 | -0.052 | -0.2977 | No |
| 69 | ALDOA |  |  | 6448 | -0.054 | -0.2991 | No |
| 70 | SUCLG2 |  |  | 6466 | -0.055 | -0.2965 | No |
| 71 | HSD17B10 |  |  | 6762 | -0.069 | -0.3248 | No |
| 72 | SDHC |  |  | 6808 | -0.071 | -0.3240 | No |
| 73 | CBR3 |  |  | 6820 | -0.072 | -0.3192 | No |
| 74 | ECI2 |  |  | 6954 | -0.078 | -0.3280 | Yes |
| 75 | GLUL |  |  | 6961 | -0.079 | -0.3220 | Yes |
| 76 | DECR1 |  |  | 6981 | -0.079 | -0.3176 | Yes |
| 77 | SUCLG1 |  |  | 7007 | -0.082 | -0.3136 | Yes |
| 78 | IDH3B |  |  | 7099 | -0.087 | -0.3168 | Yes |
| 79 | UROD |  |  | 7141 | -0.090 | -0.3140 | Yes |
| 80 | ALDH3A2 |  |  | 7250 | -0.097 | -0.3183 | Yes |
| 81 | PSME1 |  |  | 7399 | -0.108 | -0.3264 | Yes |
| 82 | PDHB |  |  | 7438 | -0.112 | -0.3214 | Yes |
| 83 | ACAA2 |  |  | 7550 | -0.122 | -0.3239 | Yes |
| 84 | CRAT |  |  | 7655 | -0.133 | -0.3247 | Yes |
| 85 | ECH1 |  |  | 7658 | -0.134 | -0.3137 | Yes |
| 86 | UBE2L6 |  |  | 7684 | -0.137 | -0.3050 | Yes |
| 87 | ECI1 |  |  | 7748 | -0.146 | -0.3001 | Yes |
| 88 | MGLL |  |  | 7773 | -0.148 | -0.2904 | Yes |
| 89 | GRHPR |  |  | 8064 | -0.196 | -0.3074 | Yes |
| 90 | UROS |  |  | 8099 | -0.203 | -0.2942 | Yes |
| 91 | PTS |  |  | 8242 | -0.238 | -0.2907 | Yes |
| 92 | MDH2 |  |  | 8251 | -0.242 | -0.2713 | Yes |
| 93 | ECHS1 |  |  | 8325 | -0.264 | -0.2575 | Yes |
| 94 | MIF |  |  | 8377 | -0.286 | -0.2394 | Yes |
| 95 | YWHAH |  |  | 8384 | -0.289 | -0.2158 | Yes |
| 96 | PRDX6 |  |  | 8410 | -0.300 | -0.1935 | Yes |
| 97 | CBR1 |  |  | 8488 | -0.334 | -0.1743 | Yes |
| 98 | PCBD1 |  |  | 8493 | -0.336 | -0.1465 | Yes |
| 99 | ODC1 |  |  | 8576 | -0.385 | -0.1236 | Yes |
| 100 | SMS |  |  | 8624 | -0.430 | -0.0929 | Yes |
| 101 | S100A10 |  |  | 8717 | -0.558 | -0.0566 | Yes |
| 102 | LGALS1 |  |  | 8744 | -0.718 | 0.0008 | Yes |
Table: GSEA details [plain text format]

  

Fig 2: HALLMARK\_FATTY\_ACID\_METABOLISM: Random ES distribution      
 Gene set null distribution of ES for **HALLMARK\_FATTY\_ACID\_METABOLISM**
